# Effects of Two-Year Vitamin B_12_ and Folic Acid Supplementation on Depressive Symptoms and Quality of Life in Older Adults with Elevated Homocysteine Concentrations: Additional Results from the B-PROOF Study, an RCT

**DOI:** 10.3390/nu8110748

**Published:** 2016-11-23

**Authors:** Elisa J. de Koning, Nikita L. van der Zwaluw, Janneke P. van Wijngaarden, Evelien Sohl, Elske M. Brouwer-Brolsma, Harm W. J. van Marwijk, Anke W. Enneman, Karin M. A. Swart, Suzanne C. van Dijk, Annelies C. Ham, Nathalie van der Velde, André G. Uitterlinden, Brenda W. J. H. Penninx, Petra J. M. Elders, Paul Lips, Rosalie A. M. Dhonukshe-Rutten, Natasja M. van Schoor, Lisette C. P. G. M. de Groot

**Affiliations:** 1 EMGO Institute for Health and Care Research, VU University Medical Center, P.O. Box 7057, 1007 MB Amsterdam, The Netherlands; e.sohl@vumc.nl (E.S.); harm.vanmarwijk@manchester.ac.uk (H.W.J.v.M.); k.swart@vumc.nl (K.M.A.S.); b.penninx@vumc.nl (B.W.J.H.P.); p.lips@vumc.nl (P.L.); nm.vanschoor@vumc.nl (N.M.v.S.); 2 Department of Epidemiology and Biostatistics, VU University Medical Center, P.O. Box 7057, 1007 MB Amsterdam, The Netherlands; 3 Division of Human Nutrition, Wageningen University, P.O. Box 8129, 6700 EV Wageningen, The Netherlands; nikitavanderzwaluw@gmail.com (N.L.v.d.Z.); janneke.vanwijngaarden@gmail.com (J.P.v.W.); elkse.brouwer-brolsma@wur.nl (E.M.B.-B.); rosalie-dhonukshe-rutten@wur.nl (R.A.M.D.-R.); lisette.degroot@wur.nl (L.C.P.G.M.d.G.); 4 Department of General Practice and Elderly Care Medicine, VU University Medical Center, P.O. Box 7057, 1007 MB Amsterdam, The Netherlands; p.elders@vumc.nl; 5 Primary Care Research Center, Institute of Population Health, University of Manchester, Oxford Road, M13 9PL Manchester, UK; 6 Department of Internal Medicine, Erasmus University Medical Center, P.O. Box 2040, 3000 CA Rotterdam, The Netherlands; a.enneman@erasmusmc.nl (A.W.E.); s.c.vandijk@erasmusmc.nl (S.C.v.D.); a.ham@erasmusmc.nl (A.C.H.); n.vandervelde@erasmusmc.nl (N.v.d.V.); a.g.uitterlinden@erasmusmc.nl (A.G.U.); 7 Department of Psychiatry, VU University Medical Center/GGZ inGeest, P.O. Box 7057, 1007 MB Amsterdam, The Netherlands; 8 Department of Internal Medicine/Endocrinology, VU University Medical Center, P.O. Box 7057, 1007 MB Amsterdam, The Netherlands

**Keywords:** homocysteine, hyperhomocysteinemia, depressive symptoms, quality of life, vitamin B_12_, folic acid, older adults, randomized controlled trial

## Abstract

Lowering elevated plasma homocysteine (Hcy) concentrations by supplementing vitamin B_12_ and folic acid may reduce depressive symptoms and improve health-related quality of life (HR-QoL) in older adults. This study aimed to test this hypothesis in a randomized controlled trial. Participants (*N* = 2919, ≥65 years, Hcy concentrations ≥12 µmol/L) received either 500 µg vitamin B_12_ and 400 µg folic acid daily or placebo for two years. Both tablets contained 15 µg vitamin D_3_. Depressive symptoms were measured with the Geriatric Depression Scale-15 (GDS-15). HR-QoL was assessed with the SF-12 Mental and Physical component summary scores and the EQ-5D Index score and Visual Analogue Scale. Differences in two-year change scores were analyzed with Analysis of Covariance (ANCOVA). Hcy concentrations decreased more in the intervention group, but two-year change scores of the GDS-15 and three of four HR-QoL measures did not differ between groups. The EQ-5D Index score declined less in the intervention group than in the placebo group (mean change 0.00 vs. −0.02, *p* = 0.004). In conclusion, two-year supplementation with vitamin B_12_ and folic acid in older adults with hyperhomocysteinemia showed that lowering Hcy concentrations does not reduce depressive symptoms, but it may have a small positive effect on HR-QoL.

## 1. Introduction

Depression is a leading cause of disability worldwide and induces substantial individual and societal burden [1]. In addition, health-related quality of life (HR-QoL) can be compromised by both mental disorders and reduced physical functioning [2,3]. Depression and low HR-QoL increase the risk of institutionalization and mortality [4]. Especially in older adults, management of depression is often suboptimal, for instance due to side-effects of antidepressant medication or interactions with other drugs [5]. Such complexities emphasize the need for simple and safe interventions for both the prevention and treatment of depression.

Elevated plasma homocysteine (Hcy) concentrations are common in older adults [6,7]. Hyperhomocysteinemia has been associated with various adverse health conditions, including cardiovascular disease [6], fractures [7], dementia [8], decreased physical functioning [9], and mortality [10]. In addition, observational studies suggest a link between elevated Hcy concentrations and depressive symptoms [11,12,13,14] and lower HR-QoL [15,16]. A meta-analysis of nine observational studies showed that persons with Hcy ≥12.5 µmol/L had a 70% higher risk of prevalent depression than persons with Hcy concentrations <12.5 µmol/L [17]. Supplementation with vitamin B_12_ and folic acid decreases Hcy concentrations [18] and may thereby reduce depressive symptoms and improve HR-QoL. However, trials investigating these effects are scarce and show inconsistent results [16,19,20,21,22,23,24]. Heterogeneity in study duration, study samples, and supplement dose may explain the observed differences.

Insufficient amounts of folate and vitamin B_12_ limit the conversion of Hcy into methionine. Methionine is a direct precursor of *S*-adenosylmethionine (SAM). SAM plays an important role in the methylation of neurotransmitters involved in depression, such as serotonin, dopamine, and noradrenalin [25,26]. In accordance with this, lower concentrations of SAM and monoamine neurotransmitter metabolites were observed in the cerebrospinal fluid of severely depressed patients who had high Hcy concentrations, compared to similar patients who did not have elevated Hcy concentrations [27].

The current amount of evidence regarding the role of Hcy, vitamin B_12_, and folic acid in depression and HR-QoL is limited. To gain more insight into these complex relationships, we investigated a large sample of older adults with mildly elevated homocysteine concentrations both cross-sectionally and after two years of supplementation with vitamin B_12_ and folic acid. It was hypothesized that Hcy is positively associated with depressive symptoms, and inversely associated with HR-QoL at baseline, and that supplementation with vitamin B_12_ and folic acid decreases Hcy concentrations, thereby reducing depressive symptoms and improving HR-QoL.

## 2. Materials and Methods

### 2.1. Study Design and Participants

Data from the B-vitamins for the PRevention Of Osteoporotic Fractures (B-PROOF) study were used for the present study. The B-PROOF study is a multi-center, randomized, parallel-group, double-blind, placebo-controlled intervention trial investigating the effect of two-year daily vitamin B_12_ (500 µg) and folic acid (400 µg) supplementation versus placebo on fracture incidence in a large sample of older adults [28,29]. Depressive symptoms and HR-QoL were predefined secondary outcome measures of the B-PROOF study.

The B-PROOF study included 2919 older adults (≥65 years) from the general population. Participants were recruited from three regions in The Netherlands: Wageningen, Amsterdam, and Rotterdam. Participants were included if they had elevated Hcy concentrations (12–50 µmol/L). Exclusion criteria included cancer diagnosis within the last 5 years (except for non-melanoma skin cancer), being bedridden, serum creatinine concentration of >150 µmol/L, current or recent (<4 months) intramuscular injections of vitamin B_12_, use of high-dose folic acid supplements (>300 µg per day), and participation in other intervention studies. Detailed information concerning recruitment, participants, and study procedures has been extensively reported elsewhere [28].

This study was conducted according to the guidelines laid down in the Declaration of Helsinki and all procedures involving human subjects were approved by the Medical Ethics Committee of Wageningen University. Local feasibility was approved by the Medical Ethics Committees of Erasmus Medical Center Rotterdam and VU University Medical Center Amsterdam. Written informed consent was obtained from all participants prior to the start of the intervention. The B-PROOF trial is registered with clinicaltrials.gov as NCT00696514 and with the Netherlands Trial Register as NTR1333.

### 2.2. Intervention

Participants were randomized in a 1:1 ratio to either the intervention group or the placebo group. Randomization was performed by an independent person by means of computer-generated numbers in stratified permuted blocks of size 4, stratified by sex, age (65–80 years, ≥80 years), study center, and Hcy concentrations (12–18 µmol/L, ≥18 µmol/L). Both groups received a daily oral tablet for a duration of two years. The tablets of the intervention group contained 500 μg vitamin B_12_, 400 μg folic acid, and 15 μg vitamin D_3_ (cholecalciferol). The placebo tablets contained 15 μg vitamin D_3_. Tablets were similar in appearance, smell, and taste. Vitamin D_3_ was added to both types of tablets to ensure adequate vitamin D concentrations, which was of importance for the primary outcome measure of the B-PROOF study: fracture incidence. At baseline and at the end of the trial, a structured interview took place, including questionnaires and physical measurements.

### 2.3. Outcomes

#### 2.3.1. Depressive Symptoms

The Geriatric Depression Scale-15 (GDS-15) was used to assess depressive symptoms at baseline and after two years of supplementation. The GDS-15 is a short version of the original 30-item GDS [30] and is a widely used instrument that displays good psychometric properties in various elderly populations [31,32]. Scores range from 0 to 15, with higher scores indicating more symptoms. A score of 5 or higher indicates the presence of clinically relevant depressive symptoms [31].

#### 2.3.2. Health-Related Quality of Life

HR-QoL is a multidimensional construct. We therefore used both the 12-item Short Form Health Survey (SF-12) [3] and the EuroQol 5 Dimensions (EQ-5D) [33] to take into account different aspects of HR-QoL (mental, physical, and general HR-QoL). Both questionnaires are self-rated, widely used, have been validated in various populations, have good test-retest reliability [3,33], and they complement each other [34].

The SF-12 is derived from the SF-36 questionnaire [35] and assesses eight health aspects: physical functioning, bodily pain, role limitations due to physical problems, general health, vitality, social functioning, role limitations due to emotional problems, and mental health. Mental (MCS) and physical (PCS) component summary scores were calculated. These scores were standardized to US general population norms [36], as Dutch norms are currently not available. Scores range from 0 to 100, with 0 representing lowest HR-QoL and 100 indicating best possible HR-QoL, with a normalized mean around 50 and a standard deviation (SD) of 10.

The EQ-5D covers five dimensions of HR-QoL: mobility, self-care, usual activities, pain/discomfort, and anxiety/depression [33]. A standardized index score (EQ-5D Index) was calculated based on Dutch norm data, ranging from −0.33 to 1, with higher scores indicating better HR-QoL [36]. Finally, the visual analogue scale of the EQ-5D (EQ-5D VAS) was used to indicate current health status on a visual scale ranging from 0 to 100, with higher scores representing better HR-QoL.

### 2.4. Baseline Characteristics

Information about marital status, highest level of completed education, alcohol intake (light, moderate, excessive/very excessive) [37], smoking habits (never, former, current), and physical activity [38] was obtained through structured questionnaires. Weight was measured using a calibrated scale. Height was measured with a stadiometer. Subsequently, body mass index (BMI) was calculated as weight/height^2^ (kg/m^2^). The Mini-Mental State Examination (MMSE) was used to assess global cognitive function (score range: 0–30) [39].

### 2.5. Biochemical Analyses

Venous blood samples were obtained from participants in a fasted state or after a restricted light breakfast. Samples were stored at −80 °C until analysis. Plasma Hcy concentrations were measured at baseline and after two years using either the Architect i2000 RS analyzer (VU University Medical Center; Abbott Diagnostics, Wiesbaden, Germany, intra-assay CV: 2%, inter-assay CV: 4%), HPLC method (Wageningen University, intra assay CV: 3.1%, inter assay CV: 5.9%), or liquid chromatography tandem mass spectrometry (LC-MS/MS) method (Erasmus Medical Center Rotterdam; CV: 3.1%). Cross-calibration showed that the outcomes of the three centers did not differ significantly. Serum folate and three markers of vitamin B_12_ status, i.e., serum vitamin B_12_, serum holotranscobalamin (HoloTC), and serum methylmalonic acid (MMA), were measured at the Erasmus Medical Center at baseline. Folate and serum vitamin B_12_ were assessed with an electrochemiluminescence immunoassay (Elecsys 2010, Roche GmbH, Mannheim, Germany) (CV folate: 5.9% at 5.7 nmol/L and 2.8% at 23.4 nmol/L; CV vitamin B_12_: 5.1% at 125 pmol/L and 2.9% at 753 pmol/L). Serum HoloTC was assessed with the AxSYM analyser (Abbott Diagnostics, Wiesbaden, Germany) (CV: <8%) and serum MMA was measured by LC-MS/MS.

### 2.6. Statistical Analyses

Data were analyzed using SPSS version 22 (SPSS Inc., Chicago, IL, USA). A two-sided *p*-value of <0.05 was regarded as statistically significant. Data are reported as *N* (%), or as median (interquartile range (IQR)). Baseline characteristics were compared between treatment groups using non-parametric Mann-Whitney tests or Pearson Chi-square tests. Participants who dropped out during the study were compared to participants who completed the study with respect to age, sex, baseline Hcy concentration, depressive symptoms, and HR-QoL.

#### 2.6.1. Cross-Sectional Analyses

The GDS-15 and HR-QoL scores were non-normally distributed, even after transformation. Therefore, baseline associations of Hcy concentrations with depressive symptoms and HR-QoL were examined using Cox regression analyses with fixed time points. In this analysis, a constant risk period was assigned (time was fixed to 1). This technique was chosen because the obtained hazard ratio from this analysis can be interpreted as a risk ratio (RR) [40]. In case of a high prevalence of the condition of interest, this is a more conservative estimate of the true effect than an odds ratio (OR). Participants with a GDS-15 score of ≥5, indicating clinically relevant depressive symptoms [31], and participants who scored in the lowest quartile of the HR-QoL measures were considered ‘cases’. It was examined whether higher Hcy concentrations were associated with a higher risk of having depressive symptoms or low HR-QoL. Analyses were performed without adjustments (crude model), adjusted for age, sex, and study center (Model 1), and additional adjustments for education level, smoking behavior, alcohol consumption, creatinine concentrations, BMI, and MMSE (Model 2). These covariates were added because of their possible influence on both the determinant (Hcy) and the outcomes (depressive symptoms, HR-QoL) [2,41,42].

#### 2.6.2. Effect Study

We performed both intention-to-treat analyses, including all participants who completed baseline and follow-up measurements, and per-protocol analyses, including all participants who were compliant with the study protocol (≥80% of tablet intake in two years). The score distributions of the outcome variables violated normality assumptions.

*Depressive symptoms—total sample*: Due to the high number of null scores on the GDS-15 in the study sample, no change scores were calculated as this would lead to interpretation difficulties. Instead, to examine whether the treatment groups differed in number of persons with depressive symptoms after two years, logistic regression analyses were performed. The dichotomized follow-up GDS-15 score (0–4: no depressive symptoms; ≥5: depressive symptoms) [31] was taken as the outcome measure and treatment group as the independent variable. The dichotomized baseline GDS-15 score was added as a covariate to control for baseline values. Age, sex, study center, and baseline Hcy were added as covariates in a second, adjusted model as randomization was stratified based on these variables.

*Depressive symptoms—subsample*: To further examine the participants with depressive symptoms, a subgroup analysis was performed. Two-year change scores were calculated for participants who scored GDS-15 ≥5 at baseline and differences in change scores between treatment groups were analyzed using Analysis of Covariance (ANCOVA), with the GDS-15 change scores as the outcome measure, treatment as the fixed between-subjects factor, and baseline GDS-15 scores as a covariate. Age, sex, study center, and baseline Hcy were added as covariates in a second, adjusted model.

*HR-QoL*: For the HR-QoL analyses, two-year change scores were calculated for the four measures of HR-QoL (SF-12 MCS, SF-12 PCS, EQ-5D Index, and EQ-5D VAS). Subsequently, ANCOVA analyses were performed, similar to the GDS-15 subgroup analyses.

For all outcome measures, predefined interaction terms of treatment with sex, dichotomous age (65–80 and ≥80 years), and dichotomous Hcy concentration (<18 and ≥18 µmol/L) were examined in the adjusted models. The Hcy cutoff value of 18 µmol/L was chosen because stratification of the participants was also performed according to this value. If interaction terms had a *p*-value of <0.10, stratified subgroup analyses were performed. In explorative post-hoc analyses, interactions of the treatment group with plasma concentrations of vitamin B_12_, HoloTC, and MMA (all indicators of vitamin B_12_ status) and folate were tested to investigate whether these baseline plasma concentrations influenced treatment effects. These variables were dichotomized at the median value (results shown in Table A1 in Appendix A).

As a final check of the effect, it was investigated whether change in Hcy concentration was associated with depressive symptoms and/or HR-QoL over time, irrespective of the treatment group. For this purpose, linear regression analyses were performed with two-year change in Hcy as the predictor and two-year change in the GDS-15 (≥5 subgroup) and HR-QoL measures as the outcome. These analyses were adjusted for baseline values of Hcy and GDS-15/HR-QoL (crude model), additionally adjusted for age, sex, and study center (Model 1), and additionally adjusted for education level, smoking behavior, alcohol consumption, creatinine concentrations, BMI, and MMSE (Model 2).

## 3. Results

### 3.1. Sample Characteristics

The baseline characteristics per treatment group are shown in Table 1. Median age of the total group was 73 years (IQR: 69–78 years) and was similar in both treatment groups. A similar number of women was included in the two groups (50%). Only HoloTC differed significantly between the groups, with the intervention group having slightly higher baseline HoloTC concentrations (median: 65, IQR: 48–86) than the placebo group (median: 63, IQR: 45–84; *p* = 0.03). The median GDS-15 score was 1 (IQR: 0–2), and 7% of the participants had a GDS score ≥5 at baseline (*N* = 200), indicating clinically relevant depressive symptoms (similar numbers in both groups). At follow-up, slightly more participants reported depressive symptoms (9%, *N* = 223, also similar in both groups). At baseline, 210 participants (7.3%) had a vitamin B_12_ deficiency (serum vitamin B_12_ < 150 pmol/L [43]) and 89 participants (3.1%) had a folate deficiency (serum folate <10 nmol/L [34]).

Compliance to the treatment was high; mean tablet intake was 90.1% in two years and 83.8% of participants had a tablet intake of ≥80% (similar in both treatment groups). The dropout percentage was 15.2% (*N* = 222) in the B-vitamin group and 13.7% (*N* = 200) in the placebo group. This group difference was not statistically significant (*p* = 0.27). Persons who dropped out were significantly older (*p* < 0.001), were more often female (*p* = 0.01), had higher baseline Hcy concentrations (*p* < 0.001), more depressive symptoms (*p* < 0.001), and lower scores on all four HR-QoL measures (SF-12 MCS: *p* = 0.03; SF-12 PCS: *p* < 0.001; EQ-5D Index: *p* = 0.02; EQ-5D VAS: *p* < 0.001) compared to persons who completed the study. Detailed information regarding dropout has been extensively reported elsewhere [29].

### 3.2. Cross-Sectional Analyses

Table 2 shows the results of the Cox regression analyses with fixed time points for the baseline association between Hcy concentrations and the GDS-15 and HR-QoL measures. The crude model showed a weak positive association between Hcy and the GDS-15, however, this association disappeared in the fully adjusted model (RR = 1.00; 95% CI: 0.96, 1.03; *p* = 0.85). For the HR-QoL measures, a similar pattern was observed, with significant crude associations between Hcy and the SF12-PCS, EQ-5D Index, and EQ-5D VAS, but after adjustment for covariates, the significance disappeared.

### 3.3. Effect Study

As expected, Hcy concentrations decreased significantly more (*p* < 0.001) in the B-vitamin group (mean two-year change: −4.4 µmol/L, SD: 3.3) compared to the placebo group (mean two-year change: −0.2 µmol/L, SD: 4.1).

#### 3.3.1. Depressive Symptoms

*Total sample*: Logistic regression analyses showed that the number of participants with depressive symptoms did not differ between the two treatment groups after two years, when controlled for baseline depressive symptoms (OR = 1.13. 95% CI: 0.83, 1.53, *p* = 0.45 in the fully adjusted model; see Table 3).

*Subsample (GDS-15 ≥ 5)*: Table 3 also presents the results of the ANCOVA analyses for the subgroup of participants with depressive symptoms at baseline (*N* = 161). The fully adjusted model did not show significant differences in two-year GDS-15 change scores between the intervention group (mean change: 1.46, 95% CI: 0.71, 2.21) and placebo group (mean change: 1.76, 95% CI: 1.05, 2.47) (*p* = 0.55). Interaction terms of treatment with age, sex, and Hcy were not significant. Explorative post-hoc interaction analyses with vitamin B_12_ and folate status predictors revealed significant interaction terms for vitamin B_12_, MMA, and folate, but stratified analyses did not show relevant differences between the intervention and placebo group (Appendix A: Table A1).

To further examine whether Hcy concentration is associated with depressive symptoms over time, linear regression analyses in the subsample with GDS-15 ≥ 5 (irrespective of treatment group) were conducted, with two-year change in Hcy as the predictor and two-year change in GDS-15 as the outcome. The crude and adjusted analyses did not reveal a significant association between the two change scores (Table 4). All per-protocol analyses yielded similar results (data not shown but available on request from the author).

#### 3.3.2. Health-Related Quality of Life

Two-year change in the mental and physical component summary scales of the SF-12 and in the EQ-5D VAS did not differ significantly between treatment groups (Table 5). The EQ-5D Index score, however, remained stable over time in the intervention group (mean change: 0.00, 95% CI: −0.01, 0.00), whereas this score slightly decreased in the placebo group (mean change: −0.02, 95% CI: −0.03, −0.01) (*p* = 0.004).

For the EQ-5D VAS, the interaction of treatment with sex was significant (*p* = 0.05). Stratified analyses, however, did not show significant differences between treatment groups in men or women (Appendix A: Table A1). Exploratory post-hoc interaction analyses with vitamin B_12_ and folate predictors did not reveal significant interaction terms.

Linear regression analyses to investigate associations over time demonstrated that change in Hcy concentration was significantly associated with change in the EQ-5D Index score (β = −0.002; SE = 0.001; *p* = 0.004) and the SF-12 PCS score (β = −0.09; SE = 0.03; *p* = 0.01) over time (Table 4). This result indicates that these physical and general aspects of HR-QoL decreased as Hcy concentrations increased over time. Results of the per-protocol analyses were similar to the intention-to-treat analyses.

## 4. Discussion

This study investigated the role of Hcy concentrations and two years of supplementation with vitamin B_12_ and folic acid on depressive symptoms and HR-QoL in a large sample of Dutch older adults with elevated Hcy concentrations. Contrary to our hypotheses, no significant cross-sectional associations between Hcy concentrations and depressive symptoms or HR-QoL were observed, when controlling for confounding variables. Furthermore, supplementation with vitamin B_12_ and folic acid did not reveal significant differences between the two treatment groups (intervention vs. placebo) on depressive symptoms and three out of four HR-QoL measures after two years. The EQ-5D Index (measuring general HR-QoL), however, remained stable over time in the intervention group and decreased slightly but significantly in the placebo group, suggesting that B-vitamin supplementation did have a small positive effect on HR-QoL. Furthermore, the two-year change in Hcy (independent of group assignment) was associated with the two-year change in the EQ-5D Index and the physical component of the SF-12. This implies that a reduction of Hcy concentrations may lead to more stable—as opposed to declining—general and possibly also physical HR-QoL over time.

The results of the present study do not suggest a role of Hcy or vitamin B_12_ and folic acid supplementation in depressive symptoms in persons with mild hyperhomocysteinemia. Several observational studies have shown associations between Hcy and depressive symptoms [44,45,46,47,48], but some studies only found significant results in persons with low folate and/or vitamin B_12_ status [45,47]. Although Hcy concentrations were elevated in all B-PROOF participants, the number of persons with a vitamin B_12_ or folate deficiency was low (7.3% and 3.1%, respectively). Nevertheless, Hintakka et al. observed an association between vitamin B_12_ and depression in persons with vitamin B_12_ concentrations within the normal range [49].

In contrast to the present results, a prospective cohort study with 521 Korean persons (over 65 years old) from the general population observed a significant association of Hcy, vitamin B_12_, and folate with incident depressive disorder over a follow-up period of 2–3 years [48]. In accordance with our results, previous trials did not observe a beneficial effect of B-vitamin supplementation on depressive symptoms either [50,51]. Supplementation dose and method, however, differed between the trials. The present study did account for some limitations of these previous studies, by including both men and women, as opposed to only men [50], and by using a long follow-up period of two years, in contrast to only four months [51].

Regarding HR-QoL, we observed a beneficial effect of B-vitamin supplementation on the EQ-5D Index score, whereas no effects were observed on the other three HR-QoL measures. The effect, however, was very small (the difference between groups was 0.02, on a scale of −0.33 to 1) and did not reach clinical relevance [52]. Comparison of the present HR-QoL results with other intervention studies is difficult due to the heterogeneity of study designs and samples. A Danish trial studied 140 adults with elevated MMA concentrations and found no effect on the SF-36 mental and physical summary scores after four weeks of vitamin B_12_ injections, but they did observe a positive effect on the general health subscale of this instrument [23]. Although these participants were not selected on the basis of their Hcy concentrations, most participants had mildly elevated Hcy concentrations. Another trial used a drink containing vitamin B_2_, B_6_, B_12_, and folic acid that significantly lowered Hcy concentrations and improved HR-QoL (assessed with the General Health Survey) already after six weeks [16]. Other trials, however, did not observe effects on HR-QoL after study periods ranging from one to three years [20,21,22,24].

The observed significant treatment effect on the EQ-5D Index score, in contrast to the SF-12, was unexpected. In relatively healthy populations such as the B-PROOF sample, the SF-12 is usually a more sensitive instrument than the EQ-5D, as the EQ-5D may display ceiling effects [34,53]. For the scoring of the EQ-5D, Dutch norms were used, whereas the scoring system of the SF-12 was based on the US population because a Dutch sample is not currently available. The EQ-5D scoring system may have been better tailored for the B-PROOF sample, which could partly explain the observed discrepancy.

To the best of our knowledge, this study with almost 3000 participants is one of the largest studies investigating the effects of vitamin B_12_ and folic acid supplementation on mental health and well-being in older persons. The randomized placebo-controlled trial design, the large study sample, and the long follow-up period are major strengths of the B-PROOF study. In this way, relationships between Hcy, depressive symptoms, and HR-QoL could be studied both cross-sectionally and after supplementation. In addition, we were able to adequately control for possible covariates. The instruments that were used for assessing depressive symptoms and HR-QoL are widely used and have satisfactory psychometric properties. Compliance to the treatment was high, which was also reflected by the significantly decreased Hcy concentrations in the intervention group compared to the placebo group.

As depressive symptoms and HR-QoL were secondary outcome measures of the B-PROOF study, relatively few participants had clinically relevant depressive symptoms (7%) and participants generally perceived a high HR-QoL. This may have reduced the power of our analyses, especially in the subgroup of participants with depressive symptoms (*N* = 161). Furthermore, it should be mentioned that vitamin D_3_ was added to both the B-vitamin and placebo tablets. Several observational and experimental studies have suggested that vitamin D supplementation reduces depressive symptoms and improves HR-QoL [54,55], which may have had an attenuating effect on our results.

By including persons with elevated Hcy concentrations, we aimed to include a sensitive sample for the effects of vitamin B_12_ and folic acid treatment. However, although elevated Hcy concentrations are common in the general older population [28], this selection may limit generalizability to the total elderly population. In addition, the restricted range of Hcy concentrations and the absence of participants with normal Hcy concentrations may have attenuated the results of the cross-sectional analyses.

Although the two-year supplementation in this study significantly lowered Hcy concentrations, our vitamin B_12_ dose of 500 µg/day may have been too low to observe an effect on depressive symptoms. A supplementation dose of at least 1000 µg/day may have been more effective [56,57]. Insufficient vitamin B_12_ status by itself has been associated with various neuropsychiatric symptoms, including depression [57]. Although we had only a low number of vitamin B_12_ deficient participants, a Finnish study showed that adult outpatients with clinical depression had a better treatment outcome when their B_12_ status was higher, even though all participants had normal or high vitamin B_12_ concentrations [49].

To further investigate the role of homocysteine in depression and HR-QoL, future research could focus on persons with hyperhomocysteinemia, low vitamin B_12_ or folate status, and on individuals with clinically relevant depressive symptoms and/or lower HR-QoL. These persons may possibly benefit more from B-vitamin supplementation. However, it should be mentioned that participants in the intervention group reported a higher incidence of cancer compared to the placebo group. This adverse effect of the B-vitamin supplementation has been reported previously [29]. Therefore, special caution is warranted in future studies with vitamin B_12_ and folic acid.

## 5. Conclusions

In conclusion, the results of the present study indicate that reducing Hcy concentrations by supplementation with vitamin B_12_ and folic acid in a generally healthy sample of older adults with mild hyperhomocysteinemia does not reduce depressive symptoms and may have a small positive effect on general HR-QoL.

## Figures and Tables

**Table 1:** Baseline characteristics of the two treatment groups (total *N* = 2919).

	Intervention (*N* = 1461)	Placebo (*N* = 1458)	*p*
**Descriptive variables:**			
Women	736 (50)	724 (50)	0.70
Age (years)	73 (69–78)	73 (69–78)	0.38
Education (years)	9 (6–15)	9 (6–15)	0.59
Study location:			0.91
WU (Wageningen)	426 (29)	431 (30)	
Erasmus MC (Rotterdam)	649 (44)	636 (44)	
VUmc (Amsterdam)	386 (26)	391 (27)	
Smoking			0.97
Current	139 (10)	142 (10)	
Former	828 (57)	821 (56)	
Never	494 (34)	495 (34)	
Alcohol use:			0.31
Light	994 (68)	972 (67)	
Moderate	417 (29)	422 (29)	
Excessive/very excessive	50 (3)	62 (4)	
Body mass index, kg/m^2^	26.7 (24.6–29.2)	26.6 (24.6–29.4)	0.65
Physical activity (kcal/day)	546 (335–823)	556 (347–831)	0.32
MMSE (score 0–30)	28 (27–29)	28 (27–29)	0.10
**Depressive symptoms/quality of life measures:**			
GDS-15	1 (0–2)	1 (0–2)	0.45
SF12 MCS	57.1 (52.3–59.8)	56.6 (51.6–59.8)	0.29
SF12 PCS	51.3 (43.8–54.2)	50.8 (42.4–54.4)	0.32
EQ-5D Index	0.86 (0.81–1.00)	0.86 (0.81–1.00)	0.84
EQ-5D VAS	80 (75–90)	80 (75–90)	0.50
**Biochemical analyses:**			
Serum folate (nmol/L)	18.8 (14.9–24.7)	18.9 (14.8–24.5)	0.53
Serum vitamin B_12_ (pmol/L)	267 (213–341)	266 (204–343)	0.27
Serum holoTC (pmol/L)	65 (48–86)	63 (45–84)	0.03
Serum MMA (µmol/L)	0.22 (0.18–0.30)	0.23 (0.18–0.31)	0.26
Plasma Hcy (µmol/L)	14.3 (13.0–16.5)	14.5 (13.0–16.7)	0.46
Serum creatinine (mmol/L)	82.0 (71.0–94.0)	82.0 (71.0–94.0)	0.59

Values are displayed as *N* (%) or median (IQR); *p* < 0.05; *N* varies slightly between variables. MMSE: Mini-Mental State Examination; GDS-15: Geriatric Depression Scale 15-item version; SF-12: 12-item Short-Form Health Survey; MCS: Mental Component Summary score; PCS: Physical Component Summary score; EQ-5D: EuroQol 5 Dimensions; VAS: Visual Analogue Scale; holoTC: holotranscobalamin; MMA: methylmalonic acid; Hcy: homocysteine.

**Table 2:** Baseline associations (risk ratios) between Hcy concentrations and the risk of having depressive symptoms (GDS-15 ≥ 5) or being in the lowest HR-QoL quartile (intention-to-treat).

	Crude Model		Model 1 ^a^		Model 2 (Fully Adjusted) ^b^	
	RR (95% CI)	*p*	RR (95% CI)	*p*	RR(95% CI)	*p*
GDS-15	1.03 (1.00, 1.07)	0.04	1.02 (0.99, 1.06)	0.26	1.00 (0.96, 1.03)	0.85
SF-12 PCS	1.04 (1.02, 1.05)	<0.001	1.02 (1.00, 1.04)	0.04	1.01 (0.99, 1.03)	0.51
SF-12 MCS	1.02 (1.00, 1.04)	0.06	1.02 (1.00, 1.03)	0.13	1.01 (0.99, 1.03)	0.33
EQ-5D Index	1.03 (1.01, 1.05)	<0.001	1.02 (1.00, 1.04)	0.02	1.02 (1.00, 1.04)	0.11
EQ-5D VAS	1.04 (1.02, 1.05)	<0.001	1.03 (1.01, 1.04)	0.01	1.02 (1.00, 1.04)	0.15

^a^ adjusted for age and sex; ^b^ additionally adjusted for study center, education level, smoking, alcohol use, creatinine, body mass index (BMI), and Mini-Mental State Examination (MMSE). *N* varies slightly between the models and variables. Hcy: homocysteine; GDS-15: Geriatric Depression Scale, 15-item version; HR-QoL: health-related quality of life; RR: Risk ratio; SF-12: 12-item Short-Form Health Survey; PCS: Physical Component Summary score; MCS: Mental Component Summary score; EQ-5D: EuroQol 5 Dimensions; VAS: Visual Analogue Scale.

**Table 3:** Effect of the treatment on depressive symptoms, analyzed with logistic regression (total sample) and ANCOVA (subsample with symptoms) (intention-to-treat).

	Baseline	Two-Year Follow-up	Model 1 ^a^			Model 2 (Fully Adjusted) ^b^		
**Logistic regression (total sample, *N* = 2588)**	*N* with GDS-15 ≥ 5 (%)	*N* with GDS-15 ≥ 5 (%)	OR ^c^ (95% CI)	*p*		OR ^c^ (95% CI)	*p*	
			1.1 (0.8, 1.5)	0.56		1.1 (0.8, 1.5)	0.45	
B-vitamins	101 (7.0)	112 (8.6)						
Placebo	99 (6.8)	111 (8.5)						
**ANCOVA (subsample GDS-15 ≥ 5, *N* = 161)**	Median GDS-15 score (IQR)	Median GDS-15 score (IQR)	Mean change (95% CI)	*F*	*p*	Mean change (95% CI)	*F*	*p*
				0.57	0.45		0.36	0.55
B-vitamins	6 (5–8)	5 (3–7)	1.4 (0.7, 2.2)			1.5 (0.7, 2.2)		
Placebo	6 (5–8)	4 (3–6)	1.8 (1.1, 2.5)			1.8 (1.1, 2.5)		

^a^ Adjusted for baseline values of the respective outcome variable; ^b^ additionally adjusted for age, sex, homocysteine, and study center; ^c^ odds ratio of having clinically relevant depressive symptoms (GDS-15 ≥ 5) after two years. GDS-15: Geriatric Depression Scale, 15-item version.

**Table 4:** Linear regression analyses of the association of the two-year change in Hcy with the two-year change in depressive symptoms and HR-QoL (intention-to-treat).

	Crude Model ^a^		Model 1 ^b^		Model 2 (Fully Adjusted) ^c^	
	β (SE)	*p*	β (SE)	*p*	β (SE)	*p*
GDS-15 (subgroup of ≥5)	0.07 (0.06)	0.27	0.05 (0.06)	0.38	0.05 (0.06)	0.43
SF-12 PCS	−0.14 (0.03)	<0.001	−0.10 (0.03)	0.003	−0.09 (0.03)	0.01
SF-12 MCS	−0.05 (0.04)	0.18	−0.05 (0.04)	0.15	−0.05 (0.04)	0.13
EQ-5D Index	−0.003 (0.001)	<0.001	−0.003 (0.001)	<0.001	−0.002 (0.001)	0.004
EQ-5D VAS	−0.15 (0.06)	0.02	−0.09 (0.06)	0.16	−0.09 0.06)	0.16

^a^ Adjusted for baseline values of homocysteine and the respective outcome variable; ^b^ additionally adjusted for age, sex, and study center; ^c^ additionally adjusted for education level, smoking, alcohol use, creatinine, BMI, and MMSE. GDS-15: Geriatric Depression Scale, 15-item version; SF-12: 12-item Short-Form Health Survey; PCS: Physical Component Summary score; MCS: Mental Component Summary score; EQ-5D: EuroQol 5 Dimensions; VAS: Visual Analogue Scale.

**Table 5:** Comparison of the two-year change scores of HR-QoL between the two treatment groups with Analysis of Covariance (ANCOVA, intention-to-treat).

			Model 1 ^a^			Model 2 (Fully Adjusted) ^b^		
	Baseline	Two-Year Follow-up	Change Scores			Change Scores		
	Median (IQR)	Median (IQR)	Mean Change (95% CI)	*F*	*p*	Mean Change (95% CI)	*F*	*p*
**SF-12 PCS (*N* = 2594)**				0.14	0.71		0.09	0.76
B-vitamins	51.6 (44.5–54.3)	51.1 (42.9–54.4)	−0.59 (−0.95, −0.23)			−0.60 (−0.95, −0.25)		
Placebo	51.5 (43.5–54.6)	50.8 (41.8–54.7)	−0.69 (−1.05, −0.32)			−0.68 (−1.03, −0.33)		
**SF-12 MCS (*N* = 2594)**				0.14	0.71		0.18	0.67
B-vitamins	57.2 (52.3–59.8)	56.8 (51.8–59.8)	−0.52 (−0.89, −0.15)			−0.53 (−0.90, −0.16)		
Placebo	56.8 (52.0–59.8)	56.6 (51.2–59.8)	−0.42 (−0.79, −0.05)			−0.42 (−0.79, −0.04)		
**EQ-5D Index (*N* = 2617)**				8.89	0.003		8.29	0.004
B-vitamins	0.86 (0.81–1.00)	0.89 (0.81–1.00)	0.00 (−0.01, 0.01)			0.00 (−0.01, 0.00)		
Placebo	0.8 (0.81–1.00)	0.84 (0.81–1.00)	−0.02 (−0.03, −0.01)			−0.02 (−0.03, −0.01)		
**EQ-5D VAS (*N* = 2612)**				0.20	0.65		0.17	0.68
B-vitamins	80 (75–90)	80 (70–90)	−1.06 (−1.73, −0.39)			−1.07 (−1.73, −0.41)		
Placebo	80 (75–90)	80 (70–90)	−1.28 (−1.95, −0.61)			−1.27 (−1.93, −0.61)		

^a^ Adjusted for baseline values of the respective outcome variable; ^b^ additionally adjusted for age, sex, homocysteine, and study center. HR-QoL: health-related quality of life; SF-12: 12-item Short-Form Health Survey, PCS: Physical Component Summary score; MCS: Mental Component Summary score; EQ-5D: EuroQol 5 Dimensions; VAS: Visual Analogue Scale.

## Appendix A

**Table A1:** Stratified analyses for significant interaction terms (stratified at the median, intention-to-treat analyses).

	*N*	Two-Year Change Scores in the Fully Adjusted Models Mean (95% CI)	*F* ^a^	*p*
**GDS-15: Stratified analyses for folate:**				
Low Folate: (≤18.86 nmol/L)			1.63	0.21
B-vitamins	38	1.87 (0.76, 2.98)		
Placebo	41	0.86 (−0.21, 1.92)		
High Folate: (>18.86 nmol/L)			3.47	0.07
B-vitamins	38	1.19 (0.17, 2.20)		
Placebo	41	2.53 (1.56, 3.51)		
**GDS-15: Stratified analyses for MMA:**				
Low MMA: (≤0.23 µmol/L)			2.70	0.10
B-vitamins	38	0.63 (−0.50, 1.76)		
Placebo	41	1.95 (0.87, 3.04)		
High MMA: (>0.23 µmol/L)			1.12	0.29
B-vitamins	38	2.28 (1.34, 3.22)		
Placebo	41	1.57 (0.67, 2.48)		
**GDS-15: Stratified analyses for Vitamin B_12_:**				
Low Vitamin B_12_: (≤266.4 pmol/L)			1.07	0.30
B-vitamins	41	2.14 (1.13, 3.16)		
Placebo	39	1.36 (0.32, 2.41)		
High Vitamin B_12_: (>266.4 pmol/L)			4.52	0.04
B-vitamins	41	0.55 (−0.60, 1.69)		
Placebo	39	2.21 (1.18, 3.24)		
**EQ-5D VAS: Stratified analyses for sex:**				
Men:			2.75	0.10
B-vitamins	657	−0.86 (−1.73, 0.02)		
Placebo	667	−1.90 (−2.76, −1.03)		
Women:			1.07	0.30
B-vitamins	645	−1.33 (−2.32, −0.33)		
Placebo	643	−0.58 (−1.58, 0.42)		

^a^ Differences between the two treatment groups over time were tested with ANCOVA, adjusted for baseline values of the respective outcome variable, age, sex, homocysteine, and study center. GDS-15: Geriatric Depression Scale 15-item version; MMA: methylmalonic acid; EQ-5D VAS: EuroQol 5 Dimensions—Visual Analogue Scale.
